# Supplementary material for: Anti-Inflammatory Actions of G-Protein-Coupled Estrogen Receptor 1 (GPER) and Brain-Derived Estrogen Following Cerebral Ischemia in Ovariectomized Rats
Source: Biology (Basel). 2023 Jan 9;12(1):99. doi: 10.3390/biology12010099 (PMC9855882; doi:10.3390/biology12010099)

Supplementary: Original Western blot images

Figure S1. G1 administration suppresses M1 phenotypic markers of microglia in hippocampal CA1 region following GCI

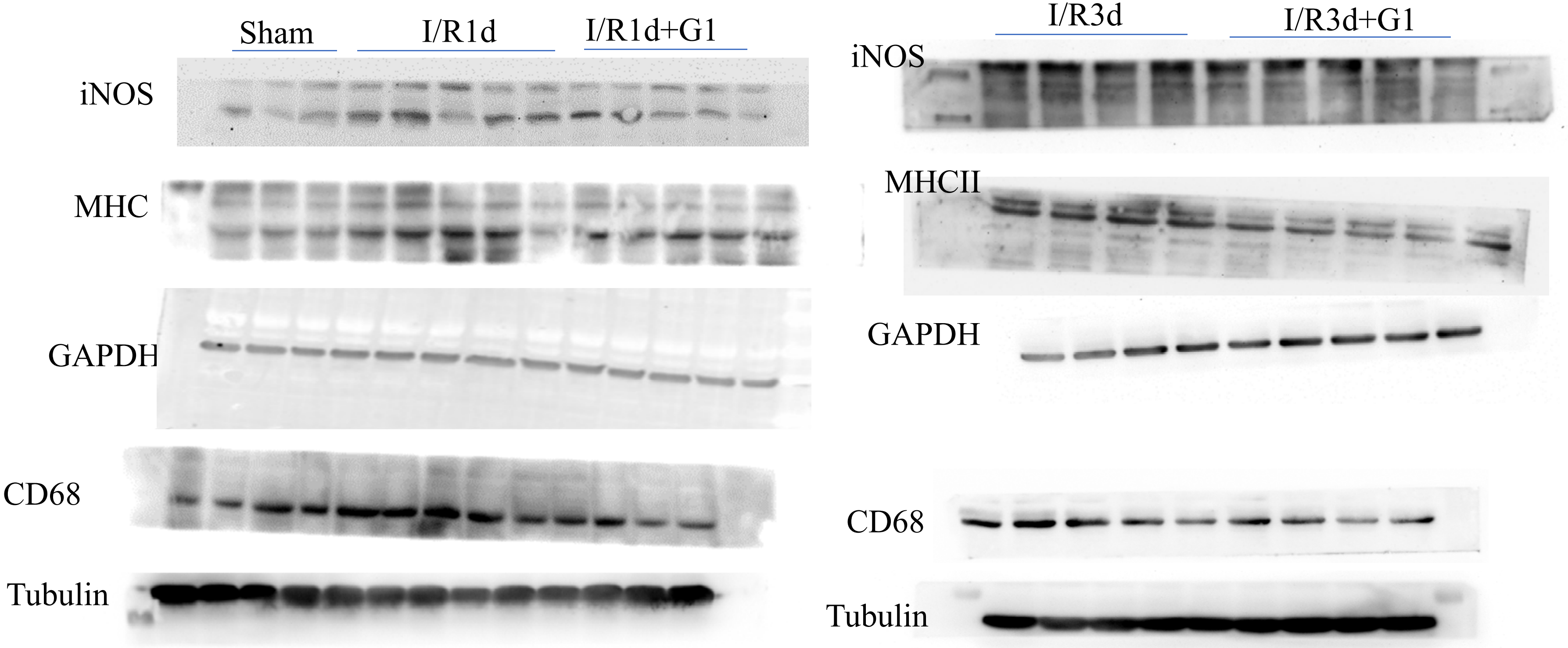

Figure S2. G1 administration enhances M2 markers of microglia in the hippocampal CA1 region following GCI

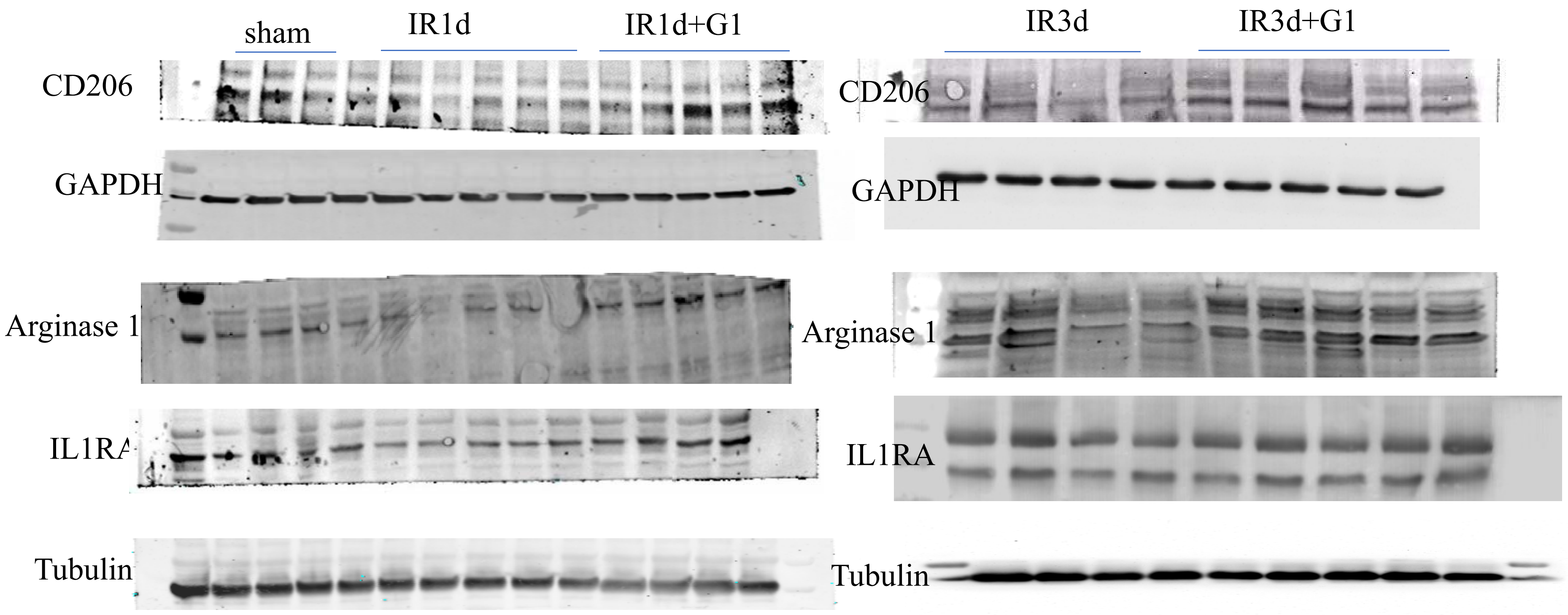

Figure S3. G1 administration increases the levels of brain-derived neurotrophic Factor (BDNF) and pro-survival transcription factor PPARγ in the hippocampal CA1 region after GCI

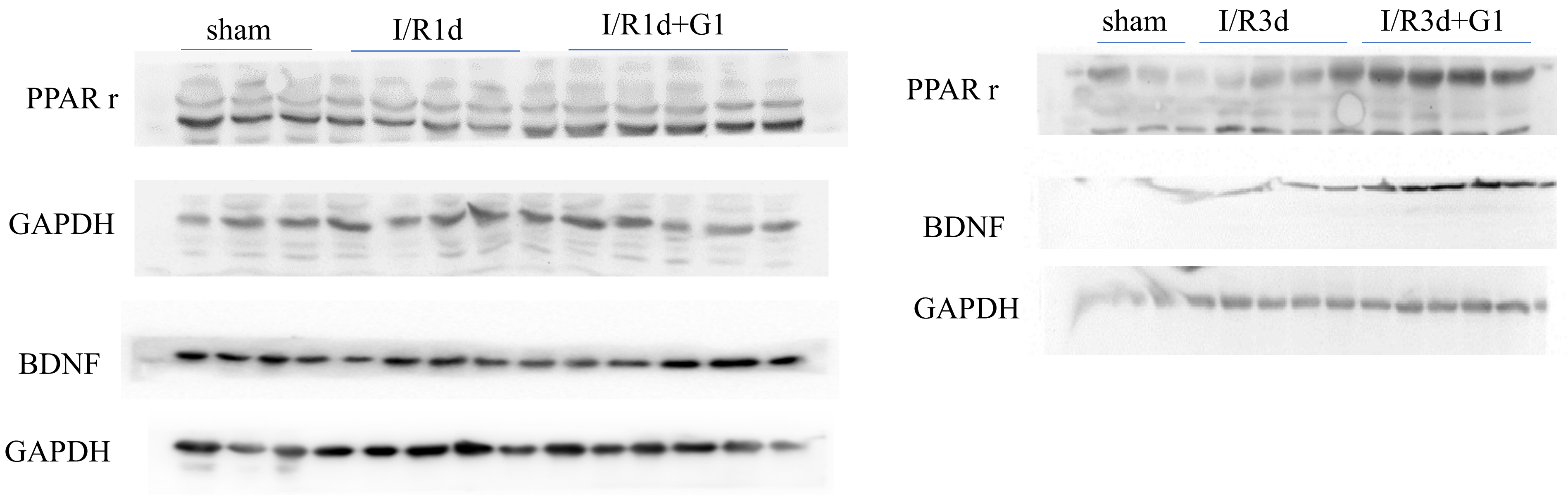

Figure S4. GPER antagonist G36 decreases aromatase protein expression in hippocampal CA1 region at 1d after GCI

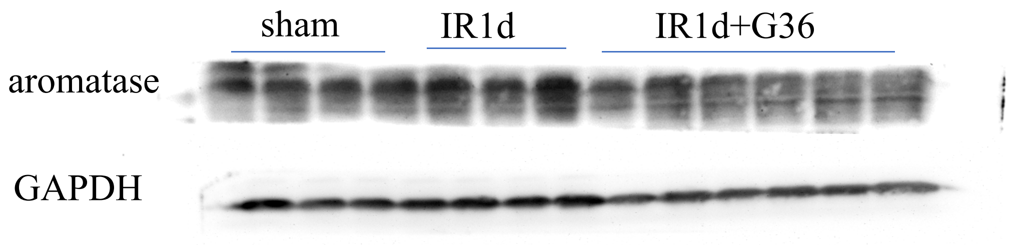

Figure S5. Aromatase inhibitor letrozole has no effect on aromatase protein levels at 1d reperfusion in hippocampal CA1 region of with and without G1-treated rats

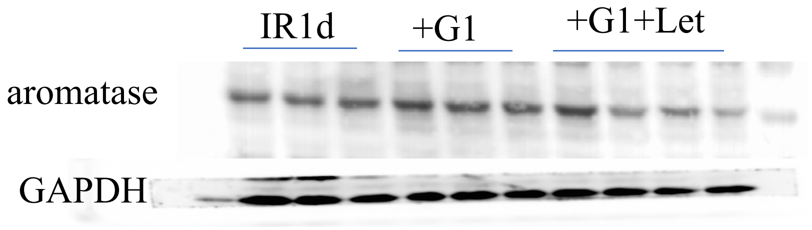

Figure S6. Using GPER antagonist G36 and aromatase inhibitor letrozole confirms the synergistic action of G1 and BDE2 in promoting protein expression of IL-4, PPARγ, and BDNF in the hippocampal CA1 region at 1d after GCI

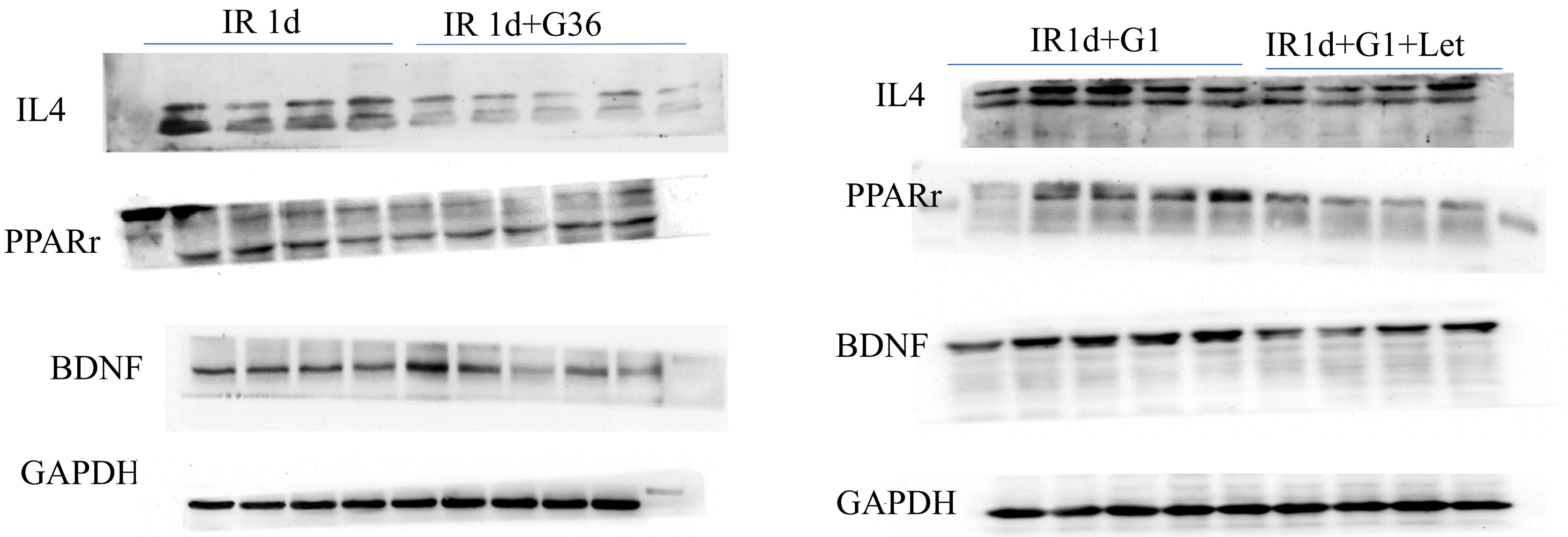

Supplement: Supplementary file 1 [file biology-12-00099-s001.zip › biology-2068044-supplementary.pdf]
